# Factors Affecting Access to Public Healthcare Facilities in the City of Tshwane, South Africa

**DOI:** 10.3390/ijerph20043651

**Published:** 2023-02-18

**Authors:** Thabiso Moeti, Tholang Mokhele, Gina Weir-Smith, Simangele Dlamini, Solomon Tesfamicheal

**Affiliations:** 1 Geospatial Analytics, eResearch Knowledge Centre, Human Sciences Research Council, Pretoria 0001, South Africa; 2 Geography, Environmental Management and Energy Studies, University of Johannesburg, Johannesburg 2006, South Africa; 3 Geography, Archaeology and Environmental Studies, Wits University, Johannesburg 2000, South Africa

**Keywords:** public healthcare access, socioeconomic factors, multivariate logistic regression, city of Tshwane, South Africa

## Abstract

Access to healthcare services is largely determined by socioeconomic factors, with economically well-off individuals obtaining healthcare services more efficiently than those who are disadvantaged. This paper aims to assess the effects of socioeconomic and other related factors on access to healthcare facilities in the City of Tshwane, South Africa, during the COVID-19 pandemic. Data were sourced from the Gauteng City-Region Observatory (GCRO) quality of life survey (2020/2021). Multivariate logistic regression was applied. Results showed that 66.3% of the respondents reported that they had access to public healthcare facilities within their area. Furthermore, results showed that those who lived in informal houses were significantly (OR = 0.55, 95% CI [0.37–0.80], *p* < 0.01) less likely to report that they had access to public healthcare facilities in their area compared to those who lived in formal houses. More efforts need to be undertaken to ensure that all citizens have access to public healthcare facilities, especially among those who are disadvantaged, such as informal dwellers. In addition, future research should encompass locality in relation to the factors that affect access to public healthcare facilities, especially during pandemics such as the COVID-19 pandemic, in order to have geographically targeted interventions.

## 1. Introduction

Primary healthcare is fundamental for improving and keeping up with the well-being of a populace [1,2]. Access to primary healthcare is a pressing research concern and policy matter in the urban setting, with a special focus on adjacent subunits [3]. Furthermore, poor access to healthcare services remains a challenge in sub-Saharan Africa [4], since access to healthcare services is largely determined by socioeconomic factors, with individuals who are economically well-off obtaining healthcare services more efficiently than those who are disadvantaged [5,6,7]. The socioeconomic status (SES) of the population and its effect on access to healthcare has not been addressed well within the South African context. Therefore, the evaluation of variation in geographical access to healthcare is important to understand the strength of a health system and to identify populations that are at high risk of preventable diseases [4]. It is therefore crucial to assess access to healthcare facilities in relation to socioeconomic and other related factors to evaluate the performance of health systems [8].

In South Africa, Christian [9] undertook a comparable study that tracked access trends in the South African public health sector from 2002 to 2012. Depending on the variables and the data’s availability, analysis was conducted at both the household and individual levels. Availability, affordability and acceptability data from the General Household Survey (GHS) were dichotomized and utilized as dependent variables in the multivariate regression models. Linear probability models (LMP) were used to analyze the data. Access to medications, fast service, travel time to public healthcare facilities and convenience of operating hours were all dependent variables for availability. Independent variables included gender, race, age, level of education, household head’s level of education, employed, household head’s employment status, real per capita expenditure and affluence. Results from the first availability LMP for the public sector revealed that there were strong and statistically significant correlations between gender, race, age, level of education and employment with drug access in the public sector [9]. The findings from this study indicated that people from the most vulnerable groups, for instance Blacks, females, the unemployed and the poor had decreased access to public healthcare facilities in terms of the availability dimension of access. Results from the affordability analyses were slightly more encouraging: access to public healthcare was more prevalent among Blacks, female, non-affluent and the unemployed. Results from the acceptability component were mixed, with Whites, men and employed people more likely to describe their experiences at public healthcare facilities as acceptable [9].

Globally, the evaluation of access to healthcare facilities continues to be the focus of both wealthy and developing countries. For example, Griffith et al. [10] evaluated how the Affordable Care Act (ACA) had altered socioeconomic gaps in healthcare access in the United States of America. The findings revealed that persons with lower socioeconomic status (SES) had significant access improvements within the first two years of the ACA’s full implementation. Arpey et al. [11] conducted a study at the University of Iowa (USA) hospital and clinics to determine how and whether patients with a low SES believe that clinical prejudice may have an impact on their healthcare. Results indicated that most respondents believed that SES had an impact on their access to healthcare. Treatment delivered, access to care and interactions between patients and doctors were common topics. In Brazil, Nunes et al. [8] assessed the link between inequalities in access, utilization and quality of healthcare services and SES. The association of socioeconomic indicators with the following factors was assessed: usage of administrations, waiting period (in days) for help and waiting time (in hours) in queues and lack of access to medical services. Analysis using Poisson regression showed that the absences of admittance to medical service administrations and holdup time in lines were higher among people of a lower financial status and that the holdup period to get care was higher among those with a higher financial status.

A similar observation can be made in Africa. For example, Saeed et al. [5] assessed the impact of socioeconomic factors on the use of various medical services by elderly men and women in Ghana. The study revealed that an older person’s health status had a significant impact on the type of healthcare service they preferred. Elderly men with higher pay favoured private healthcare services, while those with college degrees, those who self-evaluated their well-being as poor or moderate and those with health insurance favoured public healthcare services. Similarly, self-employed males and those in casual jobs preferred healthcare services that were not provided by the government. Private healthcare services were preferred by women with an elementary or secondary education. Women with health insurance, those who ranked their own health as poor or moderate, those in the middle- and upper-income quantiles, and those who were relatively young, preferred public healthcare facilities. Traditional treatment was preferred by self-employed women and those who worked part-time.

Demographic factors such as sex, population group and age influence primary healthcare accessibility [12]. Healthcare accessibility may be influenced by gender because women are seen to have more community traits than men, such as altruism and concern for others, and because female social responsibilities are more closely tied to the family than male tasks [13]. With regard to the population group, the capacity of a person to get care is influenced by race and ethnicity, which cannot simply be seen as proxies for socioeconomic issues [14]. Minority races and people with lower SES typically struggle more than people with greater SES in accessing healthcare services [15]. In the USA, Shi et al. [14] investigated racial or ethnic differences in healthcare access and found that Blacks and Hispanics were less likely than Whites to report having trouble getting access to medical, dental and pharmaceutical services. Zhang et al. [16] investigated the associations between self-reported inadequate access to care and multiple health outcomes among older men and women in China. Results showed that there was a lower accessibility of care among those aged 80 years and older and that it is sometimes linked to greater needs brought on by ill health.

Income is a fundamental indicator of social class and makes access to healthcare easier as it can be used to pay for insurance covers and doctor visits [17,18]. Okunrintemi et al. [19] assessed the variations in patient healthcare experiences due to income and indicated that patients with lower income levels have less favourable experiences with healthcare in terms of both access and care quality. In general, the health of the unemployed is worse than that of the employed because the unemployed frequently have less access to healthcare [20].

People without insurance are more likely than those with insurance to have unmet needs because they are less likely to receive doctor and preventative treatment, which eventually leads to different health outcomes and overall quality of life [21]. For example, Zhang et al. [16] reported that people aged 60 to 69 years old frequently blame a lack of insurance for their lower self-reported accessibility to healthcare. In South Africa, the National Health Insurance (NHI) is being planned to ensure that all people have equal access to healthcare. Weir-Smith et al. [22] assessed urban residents’ perceptions about the NHI implementation and factors associated to these perceptions during the early days of theCOVID-19 pandemic in South Africa. They found that those who favoured the NHI were men, those who were informally employed, people staying in informal settlements and townships, and those of the Black African and Colored populations. They also found that those respondents with matric and tertiary education were significantly less likely to be in favour of NHI.

There is a need to investigate the effects of socioeconomic and other factors on access to public healthcare facilities during the COVID-19 pandemic. The COVID-19 pandemic has dramatically highlighted the necessity for a more integrated healthcare system, and it presented a significant opportunity for NHI to be piloted in real-time [23,24]. Therefore, the current study is aimed at assessing the effects of socioeconomic and other related factors on access to public healthcare facilities in the City of Tshwane, South Africa, during the COVID-19 pandemic.

## 2. Materials and Methods

### 2.1. Data

**Figure 1:**
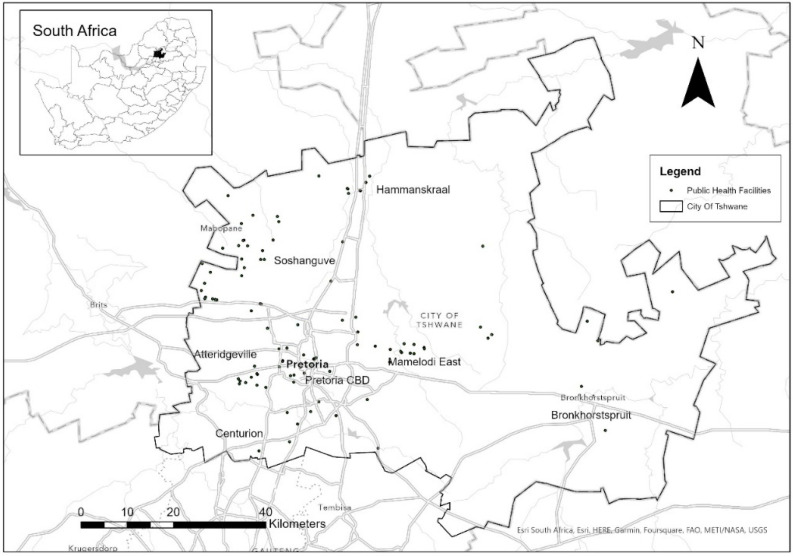
Study Area—City of Tshwane (Data source: Department of Health).

The present study utilized data from the Gauteng City-Region Observatory (GCRO) quality of life survey six (2020/2021), conducted from October 2020 to May 2021. Data were collected by trained fieldworkers using digital tablets. In addition to the training of normal data collection protocols, the fieldworkers were also trained in COVID-19 protocols as the survey was conducted during various lockdown restriction levels. The survey is conducted every two years among randomly selected adults living in households around the Gauteng province, South Africa, and is designed to be representative at a ward administrative level [25]. More details on the sampling design can be found from [25]. Benchmarking was undertaken to adjust the design weights to the newest released GeoTerraImage (GTI) 2021 population estimates, which were based on the 2020 mid-year population estimates of Statistics South Africa [26]. Benchmarking was applied for each local or metropolitan municipality in Gauteng, with population group, sex and wards as benchmark variables [26]. This process ensures that the views and perceptions of overrepresented proportions of the population are scaled down and that those less represented are illuminated [27]. Therefore, the results can be generalised to the province’s adult population. For this study, only the City of Tshwane (Figure 1) data were extracted from the province-level database. The city is divided into 107 wards with six Enumeration Areas (EAs) per ward. A minimum of four interviewees were targeted per EA, resulting in a minimum of 2782 samples; however, the final realised sample was 2810 [25]. The focus of this paper was on access to public healthcare facilities, therefore only respondents who reported that they use public healthcare facilities were considered. The resultant final sample for this paper was 1735 respondents from the City of Tshwane. The questionnaire administered in the interviews contained a total of 203 questions and consisted of two parts: the main content section that was completed by all respondents and a self-complete section that was optional and confidential. Income, which is a proxy for SES, is one of the questions that were self-completed, optional and confidential. Therefore, only 1271 respondents who utilised public healthcare facilities in the City of Tshwane completed this question. As income was included in the final multivariate logistic regression model, this meant that the final sample size for each explanatory variable included in the model was 1271, which is about three quarters of the 1735 sample.

### 2.2. Measures

The primary outcome variable, access to public healthcare facilities, was based on the question “Are there healthcare facilities you usually use in the area where you live?”, with the response options being 1 = yes and 0 = no.

Explanatory variables that were selected in the study were based on South Africa’s well-documented challenges of poverty and inequality. These variables also make vital social indicators of healthcare status assessment among the research community [8,9,10,11,12,13,14,16]. Table 1 summarizes the socioeconomic and related explanatory variables used in the study. Notice that the original categories were more than what were used in the study for most variables. This reduction in the categories was necessary to ensure a sufficient sample size per category and thereby to uphold the integrity of the statistical analysis and inferences made from the results.

### 2.3. Statistical Analysis

Statistical analyses were run in Stata software version 15.0 [28]. The “svy” command in the software was used to incorporate benchmarking weights into the analysis. A correlation matrix was performed to assess the relationship between all variables that were explored in this study. This was to ensure that there is no effect of multicollinearity on further inferential statistics [29]. None of the variables were highly correlated and thus all the variables can be used as inputs to determine their influence on healthcare service availability. The highest correlation was between income and insurance, with 0.49. To determine the relationship between one or more independent (predictor) factors and a binary dependent (outcome) variable, logistic regression is usually used [30]. Subsequently, multivariate logistic regression was used to determine factors affecting access to public healthcare facilities in the City of Tshwane, South Africa. Odds Ratio (OR) with 95% Confidence Intervals (CIs) were reported, and *p* ≤ 0.05 was considered for the level of statistical significance. 

## 3. Results

### 3.1. Characteristics of the Study Sample

The study sample comprised 1735 respondents (Table 2). After benchmarking, females constituted 53.2% and Black Africans accounted for 92.8%. Around 24.4% were 25–34 years old and 42.8% have an income between R801–R3200. The majority of people (94.6%) did not have short term insurance. Good health status was reported by 87.0% of the population and 62.6% were satisfied with their healthcare services. The majority of residents (82.3%) lived in formal housing, and 41.2% of them had lived in their area for more than 10 years.

### 3.2. Respondents That had Access to Public Healthcare Facilities within Their Area

Overall, 66.3% of the respondents reported that they had access to public healthcare facilities within their area (Table 3). Access to public healthcare facilities was higher with males (67.7%), within the population group of Black Africans (66.8%), those aged 65 and older, those who have an income between R801–R3200 (68.0%) or R25601 and more (68.1%), those who are insured (76.1%), those who perceived their health status was good (67.7%), those who are satisfied with health services (67.9%), those who are living in formal dwelling types (68.6%) and those who have always lived in their neighborhood (70.8%).

### 3.3. Factors Influencing Access to Public Healthcare Facilities

Multivariate logistics regression results showed that, as expected, White individuals (OR = 0.49, 95% CI [0.24–0.99], *p* = 0.05) were significantly less likely to report that they have access to public healthcare facilities in their area compared to Black Africans (Table 4). People who perceived that their health status in the last four weeks preceding the survey was good, were significantly (OR = 1.70, 95% CI [1.11–2.61], *p* = 0.02) more likely to indicate that they have access to public healthcare facilities in their area than those who perceived that their health status was poor. In terms of dwelling type, those who lived in informal houses were significantly (OR = 0.55, 95% CI [0.37–0.80], *p* < 0.01) less likely to have access to public healthcare facilities compared to those who lived in formal houses. For length of stay in a neighborhood, those who lived in the neighborhood for less than four years were significantly (OR = 0.62, 95% [CI = 0.39–0.99], *p* = 0.05) less likely to have access to public healthcare facilities in their area compared to those who have always lived in the neighborhood. 

## 4. Discussion

Overall, this study found that approximately 66.3% of respondents in the City of Tshwane have access to public healthcare facilities. Together with the fact that the public healthcare system supports around 84% of the South African population (those who are uninsured), these findings point to the fact that more needs to be done in terms of universal health care access. In comparison, the private healthcare sector supports only 16% of the country’s population (those with medical insurance) but has an annual per capita healthcare expenditure of almost 10 times more than that of the public sector [24,31]. The NHI seeks to guarantee that everyone living in South Africa, regardless of SES, has access to high-quality healthcare services offered by the public and private sectors [32]. Weir-Smith et al. [22] found that 77.5% of the urban residents were in favour of NHI implementation in South Africa, affirming an earlier statistic by Booysen and Hongoro [33], who indicated that more than 80% of healthcare users (from both urban and rural areas) were of the opinion that NHI was a top priority.

The current study showed that females and those living in informal dwellings are less likely to have access to public healthcare facilities. According to a comparable study that tracked access trends in the South African public health sector from 2002 to 2012, people from the most vulnerable groups, namely Blacks, females, the unemployed and the poor had decreased access to public health facilities in that period [9].

The results of this study shed significant light on availability and access to public healthcare facilities against ethnic grouping. Unsurprisingly, White populations were significantly less likely to indicate that they have public healthcare facilities in their area compared to Black Africans. This is due to the fact that the White population group reside in the suburbs or upper-class areas of the city, which are mostly dominated by private healthcare facilities, whereas there are many Black Africans located in townships which are dominated by public healthcare facilities. The analysis conducted by Shi et al. [14] revealed that Blacks and Hispanics were less likely to have difficulty accessing medical services, and these findings are comparable. This finding is strengthened by Christian’s [9] findings, which revealed that access to public healthcare was more common among black individuals and was more affordable.

When compared to people who perceived their health status as poor in the month preceding the survey, persons who perceived their health status as good were significantly more likely to indicate that there were public healthcare facilities in their area with OR = 1.73 (Table 4). Interestingly, Comber et al. [34] found that participants with poor health conditions had 1.7 times more difficulty accessing General Practitioners (GPs) in the United Kingdom (UK). In contrast to the findings from Geitona et al. [35], which indicated that those with poor health status tend to have difficulty in accessing public healthcare, the results of this study show the exact opposite pattern. Access to healthcare facilities results in better treatment, better health outcomes, the prevention of disease progression, and an improvement in quality of life, all of which contribute to good health status.

Respondents who lived in informal houses were significantly less likely to have access to public healthcare facilities within their area compared to those who lived in formal houses (Table 4). In South Africa, the demand for primary healthcare services in informal settlements is already outpacing supply due to the rising number of patients, and access to these services becomes more restricted as time goes on [36]. The majority of people living in informal settlements are usually jobless, have below-standard housing and have limited access to healthcare [36]. From the service provider’s angle, the absence of essential staff, a lack of essential drugs and supplies and a lack of oversight of how healthcare is provided were common obstacles that prevent access to the underprivileged communities [37]. Social norms are also believed to have an influence on behaviours of group members towards accessing service delivery [38,39]. For example, Prentice [40] and Diez Roux [41] stated that a person may be more likely to receive primary care if they live in a community where social norms support the adoption of healthy behaviours. Such a link between social behaviour and receiving health service is untested at least in our study area. On the contrary, it should be stressed that the only tangible evidence we have in the current study is the shortage of public healthcare facilities among disadvantaged communities.

For length of stay in a neighborhood, those who lived in the neighborhood for less than four years were less likely to have access to public healthcare facilities in their area than those who have always lived in the neighborhood. People who have always lived in the same neighborhood are more likely to be knowledgeable about the services available nearby and to have a higher level of trust in them than those who are relatively new in the neighborhood. In order to ascertain whether and when the negative acculturation theory among Asian immigrants is a suitable explanation for duration patterns, Ro [42] looked at the empirical evidence supporting it. According to Ro [42], people who have lived in the United States for a longer period of time have had more exposure to and have become more integrated into its social environment. The same may be said for neighborhoods, where people who have lived there for a longer period of time are more exposed to and assimilated to its characteristics and hence more likely to report having access to public healthcare facilities. In contrast, recently relocated residents are less likely to take full advantage of public healthcare services [43]. This has been attributed to language barriers, a lack of knowledge about the functioning of the healthcare system, lack of documentation and discrimination by healthcare professionals [44,45].

The strength of this study is that it used benchmarked data which allowed for the findings to be generalized to the City of Tshwane’s adult population. This benchmarking process also reduced some bias that could have been introduced by sampling and non-responses. One of the limitations of this study is that access to public healthcare facilities was assessed based on the question: “Are there healthcare facilities you usually use in the area where you live?”. Responses could have been underestimated or overestimated depending on the understanding of respondents on the phrase “the area where you live”. A question with a quantifiable distance such as 5km or so could have yielded more accurate results about access to public healthcare facilities than the current one. Despite this shortcoming, the findings from this paper provide significant insights in terms of factors affecting access to public healthcare facilities in the City of Tshwane, South Africa.

## 5. Conclusions

Despite the South African constitution’s promise of healthcare access for all, there are still significant disparities. Overall, this study found that 66.3% of respondents in the City of Tshwane have access to public healthcare facilities within their areas. This indicates that there is still a sizeable population who do not have access to public healthcare facilities within their neighborhood. Population group, health status, dwelling type and neighborhood length of stay were significantly associated with access to public healthcare facilities. The study showed that females and those living in informal dwellings are less likely to have access to public healthcare facilities. More effort needs to be undertaken to ensure that all citizens have access to public healthcare facilities, especially among those who are disadvantaged, such as informal dwellers. In addition, future research should encompass locality in relation to the factors that affect access to public healthcare facilities, especially during pandemics such COVID-19, in order to have geographically targeted interventions.

Improvement can be done by enhancing the calibre and accessibility of knowledge and instruction around healthcare access. Internal and cross-border referral networks, communication, and coordination mechanisms must be strengthened in order to increase migrant populations’ access to public healthcare. Moreover, primary healthcare reform programs need to include a migration-aware response as a crucial component. Having initiatives aimed at fostering closer linkages between groups of people who share similar social and cultural perspectives will help to support access to healthcare.

There should be outreach programs that can be helpful in enhancing preventative healthcare, connecting poor or disadvantaged individuals to welfare services and assisting in the establishment of support groups for these people in order to increase healthcare accessibility. Social support networks should be established by people of informal communities. To guarantee that they are provided with accessible healthcare facilities, the informal residents should be involved in the planning of healthcare provision or policy creation. Public healthcare facilities should consider offering mobile clinics or improving their frequency where they are already existing for people in informal settlements who need to access the healthcare facilities.

## Figures and Tables

**Table 1:** Socioeconomic and related variables used as inputs to determine access to healthcare facilities.

Factor	Used Categories	Original Categories
Sex	Male Female	Male Female
Population group	Black African White Other	Black Colored Indian/Asian White Other
Age	18–24 25–34 35–44 45–54 55–64 65+	18–19 20–24 25–29 30–34 35–39 40–44 45–49 50–54 55–59 60–64 65+
Income in South African Rand	1–800 801–3200 3200–12,800 12,801–25,600 >25,600	1–800 801–3200 3201–12,800 12,801–25,600 25,601–51,200 >51,200
Medical aid ownership/Insurance	Yes No Do not know	Yes No Do not know
Health status perception	Good Poor	Excellent Good Poor Very poor
Health services satisfaction	Satisfied Dissatisfied Neither satisfied nor dissatisfied	Very satisfied Satisfied Neither satisfied nor dissatisfied Dissatisfied Very dissatisfied
Dwelling type	Formal Informal/Other	Formal Informal Other
Neighborhood length of stay	Always lived here >10 years 5–10 years <5 years	I have always lived here >10 years 5–10 years 2–4 years 1–2 years <1 year

**Table 2:** Characteristics of the study sample.

	Sample	%	[95% CI]
Total	1735	100.0	
Sex			
Male	771	46.8	[44.0–49.6]
Female	964	53.2	[50.4–56.0]
Population group			
Black African	1589	92.8	[91.4–94.0]
White	105	4.8	[3.8–5.9]
Other	41	2.5	[1.8–3.4]
Age group			
18–24	244	14.1	[12.3–16.1]
25–34	395	24.4	[22.0–26.9]
35–44	371	21.5	[19.1–24.1]
45–54	301	17.8	[15.8–20.0]
55–64	222	11.9	[10.2–13.7]
65+	202	10.4	[8.8–12.2]
Income			
R1–R800	211	17.1	[14.8–19.7]
R801–R3200	557	42.8	[39.6–46.2]
R3201–R12800	387	32.5	[29.2–35.9]
R12801–R25600	75	5.2	[4.1–6.7]
R25601 and more	41	2.4	[1.7–3.4]
Insurance			
No	1623	94.6	[93.3–95.6]
Yes	104	5.1	[4.1–6.3]
Do not know	8	0.4	[0.2–0.8]
Health status			
Poor	231	13.0	[11.3–15.1]
Good	1504	87.0	[84.9–88.7]
Health services satisfaction			
Satisfied	1078	62.6	[59.9–65.3]
Neither satisfied nor dissatisfied	116	6.6	[5.3–8.1]
Dissatisfied	541	30.8	[28.3–33.4]
Dwelling type			
Formal	1445	82.3	[79.9–84.4]
Informal/Other	290	17.7	[15.6–20.1]
Neighborhood length of stay			
I have always lived here	561	32.1	[29.6–34.7]
More than 10 years	688	41.2	[38.4–44.0]
5–10 years	190	10.5	[9.0–12.3]
Less than 4 years	296	16.1	[14.0–18.6]

CI = Confidence Interval.

**Table 3:** Percentage of respondents that reported they had access to public healthcare facilities within their area.

	Sample	%	[95% CI]	*p* Value
Total	1735	66.3	[63.6–69.0]	
Sex				
Male	771	67.7	[63.4–71.6]	0.37
Female	964	65.1	[61.4–68.7]	
Population group				
Black African	1589	66.8	[63.9–69.6]	0.33
White	105	58.5	[47.1–69.1]	
Other	41	63.2	[46.4–77.3]	
Age group				
18–24	244	69.1	[61.6–75.7]	0.72
25–34	395	64.1	[58.3–69.6]	
35–44	371	67.2	[61.2–72.7]	
45–54	301	63.6	[56.9–69.8]	
55–64	222	66.5	[58.9–73.4]	
65+	202	70.5	[60.9–78.5]	
Income				
R1–R800	211	67.8	[59.9–74.8]	0.93
R801–R3200	557	68.0	[63.2–72.4]	
R3201–R12800	387	65.0	[58.5–71.0]	
R12801–R25600	75	66.8	[53.7–77.7]	
R25601 and more	41	68.1	[50.1–82.0]	
Insurance				
No	1623	65.8	[63.0–68.6]	0.15
Yes	104	76.1	[65.5–84.3]	
Do not know	8	56.9	[20.7–87.0]	
Health status				
Poor	231	57.1	[48.9–64.9]	0.01
Good	1504	67.7	[64.8–70.5]	
Health services satisfaction				
Satisfied	1078	67.9	[64.3–71.3]	0.23
Neither satisfied nor dissatisfied	116	67.0	[56.4–76.1]	
Dissatisfied	541	62.9	[58.1–67.5]	
Dwelling type				
Formal	1445	68.6	[65.6–71.4]	<0.01
Informal/Other	290	55.8	[48.7–62.6]	
Neighborhood length of stay				
I have always lived here	561	70.8	[66.3–75.0]	0.09
More than 10 years	688	65.7	[61.3–69.9]	
5–10 years	190	63.4	[55.0–71.1]	
Less than 4 years	296	60.8	[53.0–68.0]	

CI = Confidence Interval.

**Table 4:** Multivariate logistic regression model of factors affecting access to public healthcare facilities.

Health Access	OR	[95% CI]	*p* Value
Sex			
Male (ref)			
Female	0.81	[0.60–1.09]	0.16
Population group			
Black African (ref)			
White	0.49	[0.24–0.99]	0.05 *
Other	0.77	[0.33–1.78]	0.53
Age group			
18–24 (ref)			
25–34	0.74	[0.46–1.19]	0.22
35–44	0.96	[0.57–1.60]	0.87
45–54	0.61	[0.35–1.06]	0.08
55–64	0.86	[0.47–1.56]	0.62
65+	0.63	[0.32–1.24]	0.19
Income			
R1–R800 (ref)			
R801–R3200	1.01	[0.66–1.53]	0.97
R3201–R12800	0.83	[0.53–1.32]	0.44
R12801–R25600	0.99	[0.45–1.89]	0.82
R25601 and more	0.85	[0.35–2.10]	0.73
Insurance			
No (ref)			
Yes	1.26	[0.64–2.46]	0.51
Do not know	0.61	[0.15–2.49]	0.49
Health status			
Poor (ref)			
Good	1.70	[1.11–2.61]	0.02 *
Health services satisfaction			
Satisfied (ref)			
Neither satisfied nor dissatisfied	0.81	[0.45–1.44]	0.47
Dissatisfied	0.81	[0.59–1.10]	0.17
Dwelling type			
Formal (ref)			
Informal/Other	0.55	[0.37–0.80]	<0.01 **
Neighborhood length of stay			
I have always lived here (ref)			
More than 10 years	0.93	[0.65–1.34]	0.71
5–10 years	0.74	[0.45–1.22]	0.24
Less than 4 years	0.62	[0.39–0.99]	0.05 *

OR = Odds Ratio, * *p* < 0.05, ** *p* < 0.01.

## Data Availability

Available from https://www.datafirst.uct.ac.za/dataportal/index.php/collections/GCRO (accessed on 12 May 2022).
